# Identification of Flo11-like Adhesin in *Schizosaccharomyces pombe* and the Mechanism of Small-Molecule Compounds Mediating Biofilm Formation in Yeasts

**DOI:** 10.3390/microorganisms12020358

**Published:** 2024-02-09

**Authors:** Yu-Gang Zhang, Tong Zhang, Lan Lin

**Affiliations:** 1 Medical School, Key Laboratory of Developmental Genes and Human Diseases (MOE), School of Life Science and Technology, Southeast University, Nanjing 210096, China; 220213940@seu.edu.cn; 2 Department of Bioengineering, Medical School, Southeast University, Nanjing 210009, China; zhangt@sari.ac.cn

**Keywords:** *Schizosaccharomyces pombe*, Flo11-like adhesin, small-molecule compounds, indole-3-acetic acid (IAA), dodecanol, *Candida albicans*

## Abstract

Fungal infection is initiated by the adhesion of pathogens to biotic and abiotic surfaces, with various manifestations including biofilm formation and invasive growth, etc. A previous report, though devoid of functional data, speculated that the *Schizosaccharomyces pombe* glycoprotein SPBPJ4664.02 could be the homology of *Saccharomyces cerevisiae* Flo11. Here, our studies with *S. pombe* substantiated the previously proposed speculation by (1) the deletion of *SPBPJ4664.02* attenuated biofilm formation and invasive growth in *S. pombe*; (2) the *S. pombe*’s lack of *SPBPJ4664.02* could be complemented by expressing *S. cerevisiae flo11*. Furthermore, indole-3-acetic acid (IAA) and dodecanol were examined in *S. pombe* for their respective effects on biofilm formation. IAA and dodecanol at high concentrations could inhibit biofilm formation, whereas opposing effects were observed with low concentrations of these molecules. Mechanism studies with the *SPBPJ4664.02*Δ and *SPBPJ4664.02*Δ/*flo11*^OE^ versus the wild type have demonstrated that IAA or dodecanol might exert regulatory effects downstream of SPBPJ4664.02 in the signaling pathway for biofilm formation. Moreover, our research extrapolated to *Candida albicans* has pinpointed that IAA inhibited biofilm formation at high concentrations, consistent with the transcriptional downregulation of the biofilm-related genes. Dodecanol suppressed *C. albicans* biofilm formation at all the concentrations tested, in accord with the downregulation of biofilm-related transcripts.

## 1. Introduction

Human health is threatened by fungal infection. Beyond superficial fungal infections, invasive infections by fungi such as *Candida* spp. are known to impact people with compromised immunity due to medical interventions—for example, those undergoing chemotherapy therapy, those who underwent organ transplantation, and those with immunosuppressive diseases including AIDS [1]. More recently, a link has been reported between *Candida albicans* intestinal outgrowth and lasting immune activation during severe COVID-19, which may exacerbate the disease outcomes [2].

Yeast cells including non-pathogenic *Saccharomyces cerevisiae*, *Schizosaccharomyces pombe*, and pathogenic *Candida albicans*, in response to adverse environmental factors, may undergo a morphological transition from yeast to hyphal forms, the so-called filamentous growth, and, if necessary, develop into sophisticated biofilm to achieve adaptation fitness [3,4]. The morphological transition of yeasts is tightly associated with biofilm formation. The biofilm-forming ability of pathogenic yeast is recognized to be the virulence attribute as well as a major causal factor of antifungal resistance and refractory infection, thereby posing a great challenge in clinical scenarios [5,6]. Accordingly, there is in urgent need to explore and develop novel anti-biofilm strategies to fight against the increasingly emerging resistant fungal pathogens such as *Candida albicans*.

Fungal adhesion to biotic and abiotic surfaces is a crucial prerequisite for pathogen colonization and the ensuing biofilm formation [7]. The biomaterial of medical implants is appropriate for fungal cell attachment and adherence, and the utilization of indwelling devices such as catheters and prosthesis may pose a high risk for fungal biofilm infections [6,8]. The fungal adhesion process depends upon a class of cell surface glycoproteins, namely, fungal adhesins or flocculins [1]. Flo adhesins have been first discovered in brewer’s yeast *Saccharomyces cerevisiae*, among which Flo11 is the main adhesin for controlling filamentous growth, mat, and biofilm formation [7,9].

*Schizosaccharomyces pombe* is an eminent model organism for investigating yeast adhesion and biofilm formation. It has been speculated by previous studies that SPBPJ4664.02 might be *S. pombe* Flo11-like adhesin [10], based on the following facts: (1) SPBPJ4664.02 shares 55.1% of its homology with Flo11 in the functional domain through a database search; and (2) the transcripts of *SPBPJ4664.02*, via microarray analysis, were found to be upregulated in two ribosomal protein-deficient mutants of *S. pombe,* namely, *rpl32-1*Δ and *rpl32-2*Δ, in which enhanced cell flocculation was observed [11]. However, it has not been substantiated whether SPBPJ4664.02 is the functional homology of Flo11 adhesin in *S. pombe*. Thus, in this study, a gene deletion mutant of *SPBPJ4664.02* was constructed, followed by the rescue experimentation of the resulting *SPBPJ4664.02*Δ with the heterologous *flo11* gene. One of our goals is to verify that the functional role of SPBPJ4664.02 is Flo11-like.

The morphological switch from yeast to hyphal forms as well as biofilm formation are governed by many environmental cues acting through several signaling molecules in a number of yeasts, including *C. albicans* [12,13,14]. Although indole-3-acetic acid (IAA) is a well-known phytohormone in *planta*, mounting evidence has revealed the presence of IAA and its effects on the morphological changes in some yeasts [13,15,16]. In *S. cerevisiae*, IAA could induce adhesion and hyphal growth at lower concentrations, while at higher concentrations, it could inhibit the growth [13]. Apart from the small-molecule compound IAA, dodecanol, a C_12_ fatty alcohol, was demonstrated to modulate hyphal development in *C. albicans* [17,18]. Notwithstanding, the mechanisms regarding such chemical signaling molecules as IAA and dodecanol influence the yeast morphologic transition, and the biofilm formation in *S. pombe* is poorly understood. Also, how the adhesins such as Flo11-like protein (SPBPJ4664.02) and Gsf2 (galactose-specific flocculin 2, encoded by *gsf2*) interplay with chemical signaling molecules, thereby giving rise to the biofilm formation, merits in-depth investigation.

In this study, the mechanism was elucidated regarding the effects of the small-molecule compounds IAA and dodecanol on invasive (hyphal) growth and biofilm formation using *S. pombe*. The impacts of the above-described compounds on the biofilm formation of *C. albicans* were further examined. This paper represents the first report dealing with the mechanistic studies in a well-defined model of yeast *S. pombe* and subsequent extrapolating to the pathogenic yeast *C. albicans*, which is of clinical significance. Our research provides novel insight that either IAA or dodecanol have potential as the synergistic anti-film agent in combination with available antifungals to eradicate the biofilm-forming pathogenic yeast *C. albicans*.

## 2. Materials and Methods

### 2.1. Strains and Media

*Saccharomyces cerevisiae* cells were routinely grown on YPD medium (Sangon Co., Ltd., Shanghai, China). *Schizosaccharomyces pombe* cells were routinely grown on YE5S medium—5 g/L yeast extract (Oxoid, UK), 30 g/L glucose supplemented with 0.225 g/L of adenine, uracil, histidine, leucine, and lysine (Sigma, St. Louis, MO, USA). For yeast adhesion and biofilm assays, *S. pombe* wild-type, the gene-deletion mutant, and gene overexpressed strains were cultivated on LNB medium—0.067 g/L yeast nitrogen base without amino acids (Solarbio, Beijing, China), 20 g/L glucose, and salts and vitamins, as for EMM [3] and 2% *w/v* agar. The above-described yeast strains originated from our laboratory collection or were created in this study. The PCR-tagging vector pFA6a-KanMX6 used for generating the specific gene deletion mutant of *S. pombe* [19] and the GFP-tagging shuttle vector pSGP572a are gifts kindly provided by Dr. P. T. Tran, University of Pennsylvania, USA.

*Candida albicans* SC5314, a known clinical isolate from CGMCC (China general microbiological culture collection center, Beijing, China), was routinely cultured on YPD agars and, if needed, grown in RPMI1640 broth (Acmec Biochemical, Shanghai, China).

### 2.2. Construction of the SPBPJ4664.02Δ Deletion Mutant

The SPBPJ4664.02-targeting DNA fragments containing KanMX6, the so-called transformation module (Figure S1), were PCR-amplified from the genomic DNA of WT cells and plasmid pFA6a-KanMX6 using deletion primer sets (Table S1). The transformation module was introduced into a WT fission yeast cell targeting the chromosomal locus of SPBPJ4664.02 to generate the SPBPJ4664.02Δ mutant via the homologous recombination technique [20]. For details, refer to the Supplementary Methods.

### 2.3. Construction of the SPBPJ4664.02Δ/flo11^OE^

The *flo11* gene from *S. cerevisiae* BY4742 genomic DNA (gDNA) was amplified via PCR using Hi-fidelity DNA polymerase (ToYoBo, Tokyo, Japan), followed by sub-cloning into the pSGP572a vector to ensure in-frame expression with GFP under the promoter nmt1 using the primers F_BglII-*flo11* and R_Not I-*flo11* (see Table 1 for nucleotide sequences) and the ClonExpress^®^ Ultra One Step Cloning Kit (Vazyme, Nanjing, China), generating the recombinant vector pSGP572a-*flo11* (see Figure S4 in the SI for a schematic representation). Next, the *SPBPJ4664.02*Δ cells of *S. pombe* were transformed with pSGP572a-*flo11* using lithium acetate transformation, as previously described in Siam et al. [21], yielding the *SPBPJ4664.02*Δ/*flo11*^OE^ strain of *S. pombe*. Ura4^+^ transformants (positive clones) were screened on solid EMM plates, and the orientation of *flo11* inserted in the transformants was verified by colony PCR with the primers listed in Table 1. Since Flo11 is expressed in fusion with GFP, the cells of *SPBPJ4664.02*Δ/*flo11*^OE^ could be observed under confocal laser scanning microscopy (CLSM).

### 2.4. Molecular Characterization of SPBPJ4664.02Δ/flo11^OE^ for Flo11-GFP Expression

#### 2.4.1. Colony PCR of SPBPJ4664.02Δ/flo11^OE^

Transformants were grown on a solid EMM plate at 30 °C for 3 days. Four individual colonies were then selected, followed by overnight culture in YE5S for yeast proliferation. Yeast genomic DNA was obtained using the TIANamp Yeast DNA Kit (TIANGEN, Beijing, China). Colony PCR was conducted by using the primers F_nmt and R_GFP, as listed in Table 1, and the respective transformant genomic DNA as the template, where *SPBPJ4664.02*Δ was used as the negative control.

#### 2.4.2. Confocal Laser Scanning Microscopy (CLSM)

Yeast cell colonies harboring flo11-gfp fusion expression (SPBPJ4664.02Δ/flo11^OE^) were selected for overnight culture on the EMM medium. Suspensions of cells at an exponential phase were examined on a glass slide under CLSM, where the GFP was excited at 488 nm. Images were collected using an Olympus FV3000 confocal microscope with a Plan-Apo 100X/1.4 Oil objective lens, analyzed by FV31S-SW Olympus.

### 2.5. Assays of Biofilm Formation in S. pombe

The biofilm formation of fission yeast on the abiotic surface was determined using a microtiter plate-based assay, as described by Kimani et al. [22]. *S. pombe* strains of WT, gene-deletion mutants (*gsf*2Δ, *gas2*Δ, and *SPBPJ4664.02*Δ), and gene-overexpressed strains (*SPBPJ4664.02*Δ/*flo11*^OE^) were examined. Briefly, overnight cultures of *S. pombe* strains (OD_600_ 0.5, *c.a.* 10^7^ cells/mL) were harvested. One hundred microliter aliquots of the cell suspensions were transferred into 96-well flat-bottomed polystyrene plates (Corning, USA). After 8 h of cell adhesion at 30 °C, the planktonic cells were removed from each well and the plates were rinsed twice with physiological saline. After drying for 10 min, 100 µL of the LNB containing indole acetic acid (IAA) or dodecanol at different concentrations was added to each treated well. IAA and dodecanol were dissolved in 50% ethanol, giving rise to the stock solutions of 100 mg/mL and 100 μM, respectively. The control well contained the solvent (50% ethanol) of an equal volume to that used in the respective treated well as well as the growth broth lacking small-molecule compounds to make the volume 100 µL. The microplates were incubated for 72 h at 30 °C, followed by the crystal violet (CV) staining protocol to examine the biofilm cells, as described in Kimani et al. [22], where absorbance at 590 nm (A_590_) was recorded. The assays were carried out in six replicates for each treatment. The inhibition extent of biofilm formation was calculated by the following formula:

Inhibition index (%) = [A_590 (control)_ − A_590 (test)_]/A_590 (control)_ × 100%

### 2.6. The Plate-Washing Assay

This method was carried out as previously described in Cullen (2015) [23], with slight modifications. Briefly, strains of *S. pombe* of different genotypes were cultured in LNB liquid to an OD_600_ of 0.8–1.0. The cell suspension of 15 μL was patched on LNB agar with an underlying layer of YE5S agar. The plates were incubated at 30 °C for 14–21 d and photographed. Running tap water was used to wash noninvasive cells from the surface, and then the plate was photographed again (for adhesion examination). Finally, under running tap water, a gloved finger was used to rub the plate and more thoroughly remove noninvasive cells, and the plate was photographed a third time (for the filamentation test). The photographs of colonies were quantitated by densitometry using ImageJ.

### 2.7. Effects of IAA and Dodecanol on C. albicans Growth

To examine the effects of small-molecule compounds on the growth of planktonic *C. albicans*, a suspension of 2 mL at the appropriate cell density in YPD was cultivated, supplemented with IAA and dodecanol, respectively, followed by colorimetric measurement at 600 nm. Please refer to the Supplementary Methods for details.

### 2.8. Assays for the Biofilm Formation of C. albicans

The biofilm formation of *C. albicans* on polystyrene was detected by a microtiter plate-based protocol, as previously described for fission yeast *S. pombe* (refer to Section 2.5), with a slight modification. Overnight cultures of *C. albicans* strains in YPD medium were harvested. The cells were washed twice in sterile physiological saline and diluted in fresh RPMI1640 medium to a concentration of 10^6^ CFU/mL. The aliquots of cell suspensions of 100 μL were transferred into flat-bottomed 96-well polystyrene microplates (Corning, Corning, NY, USA). After incubation for 2 h at 37 ℃, the planktonic cells were removed from each well and the plates were rinsed twice with physiological saline. After drying for 10 min, 100 µL of the RPMI1640 medium containing IAA or dodecanol at different concentrations was added to each treated well. The control well contained the solvent of an equal volume to that used in the respective treated well as well as growth broth lacking small-molecule compounds to make the volume 100 µL. The plates were then incubated for 48 h at 37 ℃. For biofilm formation detection, the previously described crystal violet staining was used, in which A_590_ values served as the endpoints of biofilm formed on 96-well microplates [22,24] The assays were carried out in six replicates for each treatment.

### 2.9. Quantitative Real-Time Polymerase Chain Reaction (qRT-PCR)

Overnight cultures of fresh *C. albicans* SC5314 in YPD were collected, centrifuged at 1000× *g* for 3 min, and washed with sterile physiological saline and then resuspended in RPMI 1640 medium to a final concentration of 10^6^ CFU/mL. The cell suspensions of 2 mL were inoculated to each polystyrene flat-bottomed six-well plate and incubated at 37 °C for 2 h. The suspensions were pipetted out, followed by gentle washing of the wells with sterile physiological saline twice to remove non-adhesive cells. Subsequently, the respective well was supplemented with 1000 μg/mL IAA or 200 μM dodecanol in 2 mL of RPMI 1640, and RPMI 1640 medium was only used as the negative control, followed by incubation at 37 °C for 48 h. After the incubation, the upper medium of each well was discarded, the wells were washed twice with physiological saline. The biofilm of each well was scraped off, suspended in 1 mL of physiological saline, and transferred to a 1.5 mL Eppendorf (Ep) tube. The samples were centrifuged and rinsed with sterile water twice. The RNA was extracted from the biofilm samples in each Ep tube using the Spin Column Yeast Total RNA Purification Kit (Sangon Biotech, Shanghai, China), and cDNA synthesis was conducted using PrimeScript™ RT Master Mix (TaKaRa, Kusatsu City, Japan).

qPCR analyses of the *C. albicans* biofilm-related genes *hwp1, ece1*, and *als3* were performed using TB Green^®^ Premix Ex Taq™ II (TaKaRa, Japan), according to the manufacturer’s manual [25], with the primers listed in Table S2, where the β-actin gene *act1* was used as an internal control to standardize the transcripts of the genes. All analyses were conducted in the Applied Biosystems StepOnePlus Real-Time PCR System in triplicate for each sample.

### 2.10. Statistical Analysis

In this study, the significant differences between treated and untreated samples were determined by Student’s *t*-test, where *p* < 0.05 was considered as statistical significance and indicated by asterisks.

## 3. Results and Discussion

SPBPJ4664.02 is a member of fission yeast *S. pombe* glycosylphosphatidylinositol-modified (GPI) glycoproteins in association with the adhesion of fission yeast cells [26]. To test the hypothesis that SPBPJ4664.02 may be the fission yeast homology of Flo11, a known adhesin of the brewer’s yeast *S. cerevisiae* [7], our study examined the biofilm-forming abilities and filamentation assays following the genetic construction of the *SPBPJ4664.02*Δ mutant strain (see Supplemental Information) and the subsequent complementation experiment by introducing the heterologous (*S. cerevisiae*) *flo11* gene into the *SPBPJ4664.02*Δ of fission yeast, in comparison to wild-type (WT) fission yeast.

### 3.1. Rescue of the SPBPJ4664.02Δ Mutant by flo11 Overexpression

**Figure 1:**
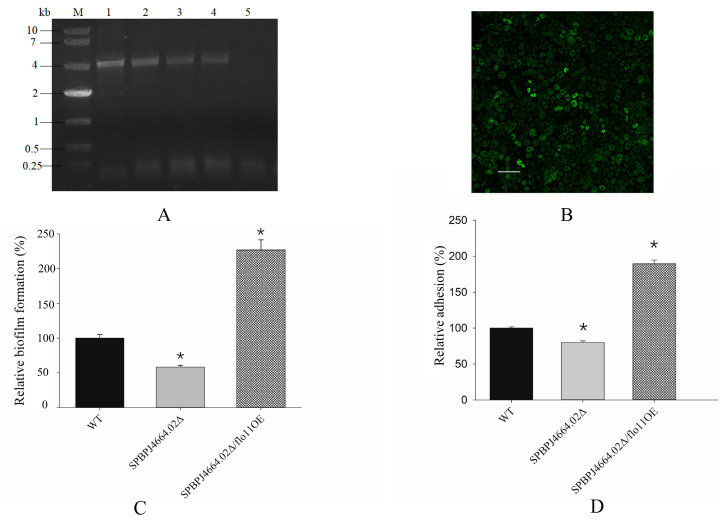
Complementation of fission yeast *SPBPJ4664.02*Δ by heterologous *flo11*. (**A**) Validation of the *flo11* in the transformants by colony PCR followed by 0.8% agarose gel electrophoresis. M: DNA ladder; #1–4 indicate the PCR products of *SPBPJ4664.02*Δ/*flo11*^OE^ transformants 1–4, respectively, of which the bands reflected as expected. Sample #5 indicates the negative control. (**B**) Flo11-GFP was co-expressed in the interior and periphery of *SPBPJ4664.02*Δ/*flo11*^OE^ fission yeast cells, as examined under CLSM. Scale bar, 10 µm. (**C**) Assays of biofilm formation in *S. pombe* WT, *SPBPJ4664.02*Δ, and *SPBPJ4664.02*Δ/*flo11*^OE^, respectively. *n* = 6 for each genotype. (**D**) Adhesion determined in three aforementioned genotypes of *S. pombe*, showing the representative illustration. *n* = 3 for each genotype. In the bar graphs (**C**,**D**), black: WT; grey: *SPBPJ4664.02*Δ; net: *SPBPJ4664.02*Δ/*flo11*^OE^. The asterisk * indicates significant differences (*p* < 0.05) using Student’s T test.

By introducing the *flo11* gene of *S. cerevisiae* origin into *SPBPJ4664.02*Δ fission yeast cells with the aid of pSGP572a-*flo11*, the transformants of *SPBPJ4664.02*Δ/*flo11*^OE^ were obtained on EMM plates, followed by colony PCR identification (Figure 1). Four transformants were selected, and *SPBPJ4664.02*Δ was used as the negative control. Colony PCR showed the successful transformation of *SPBPJ4664.02*Δ cells with the recombinant vector pSGP572a-*flo11* (Figure 1A).

Since *flo11* was co-expressed with *gfp* under the Pnmt1, the expressed GFP was examined under CLSM. Notably, the Flo11-GFP fusion protein was expressed in the interior and periphery of *SPBPJ4664.02*Δ cells under CLSM (Figure 1B).

In the biofilm assay (Figure 1C), the biofilm-forming ability of different genotypes of *S. pombe* was determined. The extent of biofilm formed in *SPBPJ4664.02*Δ decreased by 40% as compared to WT (*p* < 0.05), indicating that *SPBPJ4664.02* is required for biofilm formation. In addition, as shown in Figure 1C, *SPBPJ4664.02*Δ/*flo11*^OE^ tremendously restored the biofilm formation ability manifested by *SPBPJ4664.02*Δ in comparison to WT (*p* < 0.05), which clearly demonstrated that *flo11* is involved in biofilm formation as well as that the expression of *flo11* might compensate for the deficiency of *SPBPJ4664.02*.

The agar adhesion abilities of different fission yeast genotypes were also tested. As shown in Figure 1D, *SPBPJ4664.02*Δ was found to form less adhesion than WT (*p* < 0.05), whereas *SPBPJ4664.02*Δ/*flo11*^OE^ restored the adhesion (*p <* 0.05) that was compromised in *SPBPJ4664.02*Δ. The results not only indicated the involvement of *SPBPJ4664.02* and *flo11* in the adhesion but also implied that *flo11* overexpression could make up for the impairment of *SPBPJ4664.02*.

Altogether, the gene deficiency of *SPBPJ4664.02* in the *SPBPJ4664.02*Δ mutant of fission yeast appeared to be complemented by expressing the *S. cerevisiae*-originated *flo11*, suggesting the functional similarity of *SPBPJ4664.02* and *flo11*, which is reminiscent of the previous studies of Watson and Davey [27] and Younes and Khalaf [28]. Taking into account of the consensus sequence of SPBPJ4664.02 sharing with Flo11 via BLAST searches and the upregulated transcripts of *SPBPJ4664.02* in *rpl32-1*Δ and *rpl32-2*Δ, the two ribosomal protein-deficient mutants of *S. pombe* concomitant with enhanced cell flocculation [11], it is strongly indicated that *SPBPJ4664.02* is the fission yeast homology of *flo11*.

### 3.2. Impacts of SPBPJ4664.02 (Flo11-like) in the Small-Molecule Signaling Pathway for Biofilm Formation and Filamentation

#### 3.2.1. Effects of IAA and Dodecanol on *S. pombe* Biofilm Formation and Cell Adhesion

Previous studies have shown that the small-molecule compound IAA at low concentrations could induce the adhesion and filamentation of the budding yeast *S. cerevisiae*, whereas at high concentrations, the inhibitory effects of IAA were observed [13]. Dodecanol is a structural analogue of farnesol, a known quorum sense (QS) signaling molecule that inhibits *S. cerevisiae* filamentation (viz. morphologic transition) and likewise in the case of *C. albicans* [29,30]. Since morphologic transition is a prerequisite for the yeast biofilm formation [4], it is hypothesized that the above-mentioned compounds, IAA and dodecanol, could be involved in the formation of yeast biofilm, likely via the dose-dependent mode. In this study, the effects of different levels of IAA and dodecanol on the biofilm formation and cell adhesion in fission yeast were investigated.

**Figure 2:**
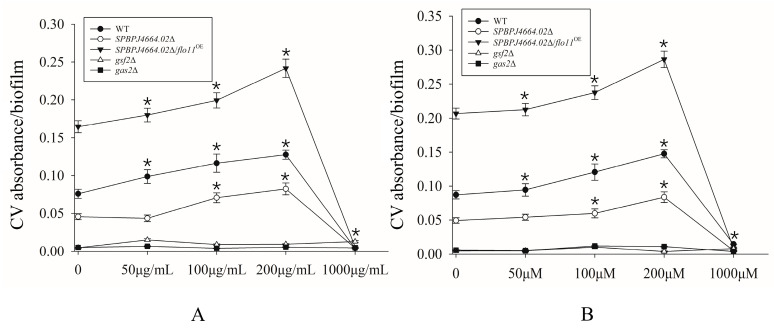
Effects of different levels of small-molecule compounds on the biofilm formation of fission yeast *S. pombe*, as determined by a crystal violet (CV) staining assay. IAA (**A**) and dodecanol (**B**) at different concentrations affecting biofilm formation. The asterisk * indicated significant differences (*p* < 0.05) using Student’s T test.

It is widely recognized that the genes *gsf*2 (coding the galactose-specific flocculin) and *gas2* (coding the cell wall remodeling enzyme) are both involved in the flocculation of *S. pombe* [31,32]. When IAA or dodecanol was added, the results indicated that the effect of small-molecule compounds on biofilm formation was dose-dependent within a certain concentration range (Figure 2). The formation of biofilms was observed to elevate in *SPBPJ4664.02*Δ, *SPBPJ4664.02*Δ/*flo11*^OE^, as well as WT fission yeasts upon the supplementation of IAA at concentrations less than 200 μg/mL. Similarly, promotive effects of dodecanol (<200 μM) on the biofilm formation were found in the *SPBPJ4664.02*Δ, *SPBPJ4664.02*Δ/*flo11*^OE^, as well as WT fission yeasts. In contrast, at high concentrations (for example, 1 mg/L IAA or 1 mM dodecanol), *c.a.* 90% of the tested yeasts displayed suppressive biofilm formation (*p* < 0.05). Neither *gsf*2Δ nor *gas*2Δ exhibited biofilm formation at the tested concentrations of IAA and dodecanol. The fact that *gsf*2Δ or *gas*2Δ biofilm formation was unaffected by either of IAA and dodecanol indicated that IAA or dodecanol molecules might act independently of Gsf2 (another flocculin) and Gas2 (the cell wall remodeling protein) in the signaling pathways for biofilm formation. Even in the untreated controls (no IAA or dodecanol addition), biofilm formation was barely found, suggesting that *gsf*2 and *gas*2 are indispensable to the biofilm formation process. Taking into account that fission yeast *gsf*2 encodes a kind of flocculins, the knock-out of *gsf*2 would abolish the cell flocculation, thereby blocking biofilm formation. Given that *gas2* encodes 1,3-β-glucanosyltransferase, the deletion of *gas2* would annihilate the cell wall architecture and thus cell flocculation and adhesion, thereby retarding biofilm formation.

#### 3.2.2. Effects of IAA and Dodecanol on *S. pombe* Invasive Growth

**Figure 3:**
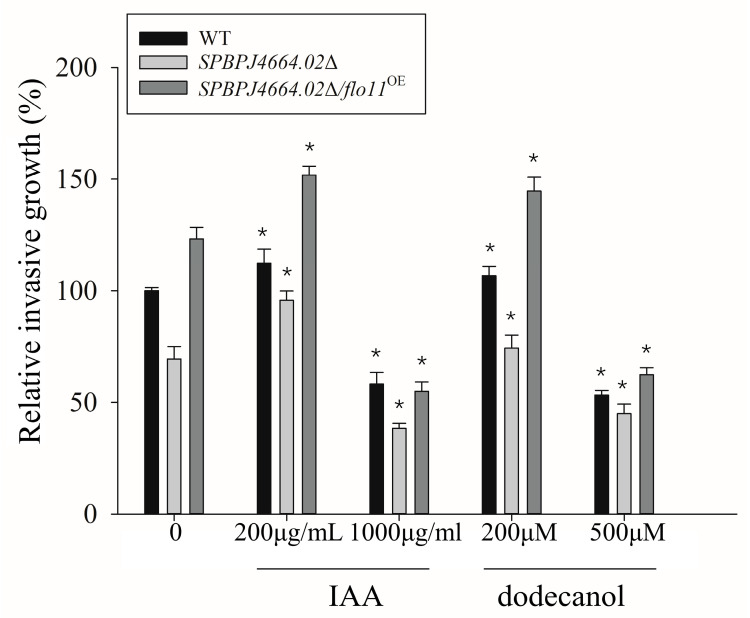
Filamentation (invasive growth) of fission yeast influenced by small-molecule compounds. Cell suspensions of 15 μL with the same cell density were spotted on LNB agar plates and cultured at 30 °C for 21 d, according to the protocol described in Section 2.6. The asterisk * indicated significant differences (*p* < 0.05) using Student’s T test.

As hyphal growth could have implications for certain important pathogenic yeasts, studying morphological transitions would provide considerable insights into pathogenicity and drug resistance. On this basis, the effects of different concentrations of IAA and dodecanol on the invasive growth (filamentation) of WT, *SPBPJ4664.02*Δ, and *SPBPJ4664.02*Δ/*flo11*^OE^ *S. pombe* were investigated. As shown in Figure 3, IAA and dodecanol at low concentrations, at 200 μg/mL and 200 μM, respectively, could promote the invasive growth to some extent, while at high concentrations, the invasive growth of *S. pombe* was remarkably inhibited. No relative invasive growth was obtained for *gsf2*Δ and *gas2*Δ because none exhibited filamentation, as previously explained in Section 3.2.1.

The biofilm formation and filamentation of *SPBPJ4664.02*Δ were influenced by IAA or dodecanol and the two traits changed with the concentration of small molecules, but they never reached the manifestation degrees of WT in terms of biofilm formation and filamentation. Consequently, we postulate that the small molecules and SPBPJ4664.02 operate in the same signaling pathway, in which small molecules likely act in the downstream of SPBPJ4664.02. Our mechanism studies with *S. pombe* herein revealed that small molecules, namely, IAA and dodecanol, might exert actions at the site downstream of Flo11-like adhesin SPBPJ4664.02 in the signaling pathway towards filamentation and biofilm formation, which represents one of the virulence traits as well as resistance in pathogenic fungi such as *Candida albicans*.

### 3.3. Effects of IAA and Dodecanol on C. albicans Biofilm Formation

**Figure 4:**
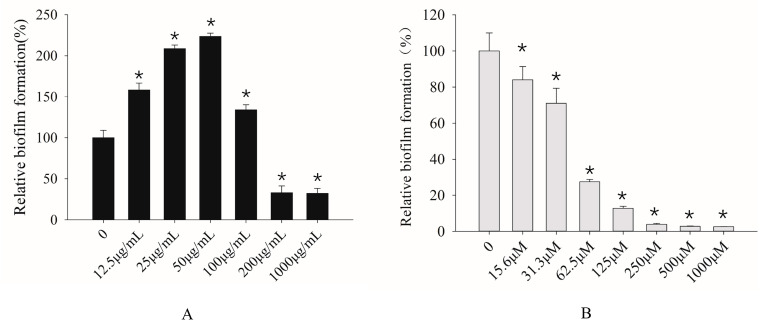
Effects of different concentrations of IAA (**A**) and dodecanol (**B**) on the biofilm formation of *C. albicans*. The asterisk * indicated significant differences (*p* < 0.05) using Student’s T test.

Given the influence of IAA and dodecanol on the biofilm formation of fission yeast, the effects of small molecules were further investigated on the biofilm formation in *C. albicans* SC5314, which is of clinical significance [33]. For IAA, it promotes biofilm formation in *C. albicans* at low concentrations while inhibiting biofilm formation at high concentrations (such as 200 μg/mL and 1000 μg/mL) by over 60% as compared to untreated counterparts (*p* < 0.05) (Figure 4A). Unlike IAA, dodecanol suppressed the biofilm formation of *C. albicans* by more than 70% in comparison to untreated counterparts (*p* < 0.05), with the minimal inhibition concentration as 62.5 μM (Figure 4B). The inhibitory impacts of dodecanol on biofilm formation showed a dose-dependent manner.

### 3.4. Transcriptional Analysis of C. albicans Biofilm-Related Genes

**Figure 5:**
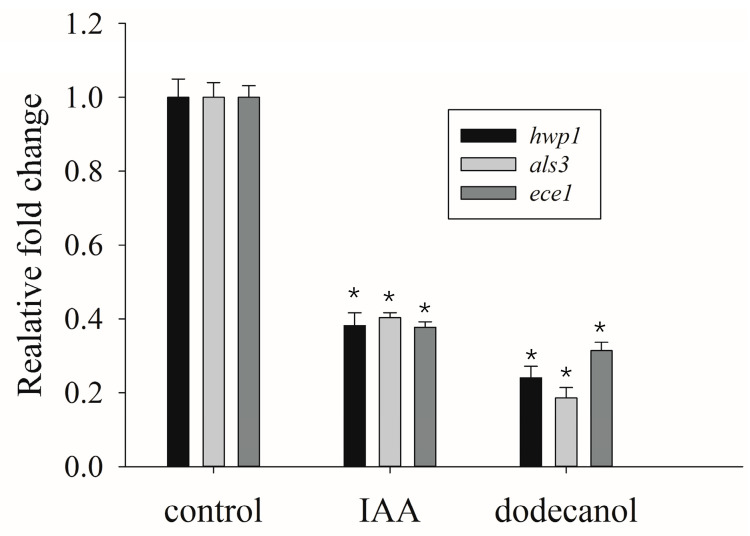
Impacts of IAA and dodecanol on the transcriptional levels of biofilm-related genes. Gene transcriptional levels were assayed in the presence of 1000 μg/mL of IAA and 200 μM of dodecanol and are presented as fold changes relative to the untreated control. The asterisk * indicated significant differences (*p* < 0.05) using Student’s T test.

In *C. albicans*, the *hwp1* (hyphal wall protein 1) and *ece1* (extent of cell elongation 1) are hyphal-specific genes, while *als3* (agglutinin-like sequence 3) is an adhesion-related gene, all of which are involved in filamentation and biofilm formation [25]. In this experiment, *act1* was used as the house-keeping gene, and untreated samples served as the control group. Accordingly, the transcriptional levels of *hwp1*, *ece1*, and *als3* were analyzed in *C. albicans* using qRT-PCR upon the treatment of small-molecule chemicals. The results revealed that 1000 μg/mL IAA significantly decreased the mRNA levels of *hwp1*, *als3*, and *ece1* by 62%, 60%, and 63%, respectively (Figure 5). Similarly, 200 μM of dodecanol was found to cause the downregulation of *hwp1*, *ece1*, and *als3* transcripts by 76%, 82%, and 70%, respectively (Figure 5).

## 4. Conclusions

In sum, the results demonstrated that the *S. pombe* protein SPBPJ4664.02 is the homology of Flo11, a known adhesin in brewer’s yeast *S. cerevisiae* governing the cell adhesion to the abiotic surface of substrates, based on the *SPBPJ4664.02* gene knock-out and complementation experimentation in combination with functional tests. *S. pombe* SPBPJ4664.02 is thereafter designated as a Flo11-like protein. Adhesion is the prerequisite for such morphological changes as filamentous growth, biofilm formation, or cell flocculation in yeasts. Analogous to Flo11 in *S. cerevisiae*, Flo11-like protein SPBPJ4664.02 was found to be required for biofilm formation as well as filamentation (evidenced by invasive growth into agar). Furthermore, our studies uncovered that the supplementation of small-molecule compounds at high concentrations such as 1000 μg/mL (IAA) or 1 mM (dodecanol) inhibited the biofilm formation in all three genotypes of *S. pombe*, namely, *SPBPJ4664.02∆*, *SPBPJ4664.02∆*/flo11^OE^ (*SPBPJ4664.02* deficiency rescued by *flo11* expression), as well as isogenic WT, though opposing effects were observed with IAA and dodecanol at low concentrations. IAA or dodecanol, as signaling molecules, exert regulatory actions probably at the downstream of Flo11-like adhesin SPBPJ4664.02 in the chemical signaling pathway for biofilm formation; however, they might act independently of Gsf2 (galactose-specific flocculin), which is indispensable for cell flocculation and thus biofilm formation. The regulatory roles of IAA or dodecanol are deduced from our investigation of *SPBPJ4664.02*Δ and *SPBPJ4664.02*Δ/*flo11*^OE^ versus isogenic WT, along with two other mutants, *gsf2*∆ and *gas2*∆ (deficient in the cell wall-remodeling enzyme), in both of which the biofilm-forming abilities were unaffected by the supplementation of physiologically relevant levels of IAA or dodecanol. Moreover, the impacts of IAA and dodecanol were further extrapolated to the medically important yeast *C. albicans*, which indicates that: (1) dodecanol had suppressive effects on biofilm formation at all concentrations tested herein; (2) IAA, administrated at high levels such as 200 and 1000 μg/mL, might inhibit biofilm formation, whereas the opposite effects were observed with IAA at low levels (12.5~100 μg/mL). Taken together, high concentrations of IAA or dodecanol could inhibit the biofilm formation in two different yeasts, *S. pombe* and *C. albicans*. Therefore, IAA and dodecanol have great potential to be developed as new anti-biofilm agents in synergy with antifungal drugs in the prevention and treatment of *C. albicans* infection, including relapsed and refractory candidiasis.

## Figures and Tables

**Table 1:** The PCR primers for generating the *SPBPJ4664.02*Δ/*flo11*^OE^ used in this study.

Application	Name of Primers	Sequence (5′-3′)
PCR of the gene *flo11* amplified from the gDNA of *S. cerevisiae*	F_Bgl II-*flo11*	5′CGCTTTGTTAAACTCGAGAGATCTCGCTTTGTTAAACTCGAGAGATCT3′
	R_Not I-*flo11*	5′TCTTCTCCTTTACTCAAGCGGCCGCGGAATACAACTGGAAGAGCGAGTAGCAACCA3′
Colony PCR of *SPBPJ4664.02∆*/*flo11*^OE^	F_nmt	5′TTCGGCAATGTGCAGCGAAAC3′
	R_GFP	5′CCTTCACCCTCTCCACTGACAGA3′

## Data Availability

Data are contained within the article and Supplementary Figures S1–S7.

## Supplementary Materials

The following supporting information can be downloaded at: https://www.mdpi.com/article/10.3390/microorganisms12020358/s1, Figure S1: Schematic representation of the transformation module, the DNA fragments for trans-forming WT *S. pombe* cells to generate *SPBPJ4664.02*Δ; Figure S2. The electrophoretic gel image of transformation module (DNA fragments targeting to *SPBPJ4664.02*) used for generating *SPBPJ4664.02*∆ mutant of fission yeast based on homologous recombination. M indicates DL2000 DNA ladder; S1–S6 indicate the products of PCR amplification using 10× diluted pFA6a-KanMX6 DNAs as the respective template; Figure S3. The molecular characterization via the colony PCR of the 3 transformants grown on the selective YE5S/G418 plates. M indicates DL2000 ladder; S1–S3 indicate the products of PCR amplification using gDNA derived from the transformants 1–3 as the template, respectively; Figure S4. Schematic representation of recombinant vector pSGP572a-*flo11*, the construct of which involves the insert of *flo11* into the Bgl II and NotI sites of pSGP572a, a shuttle vector harboring Pnmt, GFP, Ura4^+^ and pUC119 between *E. coli* and *S. pombe*; Figure S5: Effects of different concentrations of IAA on growth of *C. albicans.* The asterisk * indicated significant differences (*p* < 0.05) using Student’s T test; Figure S6: Effects of different concentrations of dodecanol on growth of *C. albicans.* The asterisk * indicated significant differences (*p* < 0.05) using Student’s T test; Figure S7: Melting curve for *act1*, *hwp1*, *ece1*, *als3* genes during the qPCR analysis; Table S1: The PCR primers for generating deletion mutant used in this study; Table S2: qPCR primer sequence.
