# Supplementary material for: Identification of Flo11-like Adhesin in Schizosaccharomyces pombe and the Mechanism of Small-Molecule Compounds Mediating Biofilm Formation in Yeasts
Source: Microorganisms. 2024 Feb 9;12(2):358. doi: 10.3390/microorganisms12020358 (PMC10893080; doi:10.3390/microorganisms12020358)
Supplement: Supplementary file 1 [file microorganisms-12-00358-s001.zip › microorganisms-2827339-supplementary.pdf]

Supplemental Information

Identification of Flo11-like adhesin in *Schizosaccharomyces pombe* and the mechanism of small-molecule compounds mediating biofilm formation in yeasts

Zhang Yu-Gang[1], Zhang Tong[2, †], Lin Lan [1,*]

1.Medical School, Key Laboratory of Developmental Genes and Human Diseases (MOE), School of Life Science and Technology, Southeast University, Nanjing 210096, China

2. Department of Bioengineering, Medical School, Southeast University, Nanjing 210009, China

* For correspondence, email: linl04@seu.edu.cn

† Current address: Building #2, 99 Haike Road, Zhangjiang Hi-tech Park, Shanghai Advanced Research Institute, Chinese Academy of Sciences, Shanghai 201210, China.

Supplemental Figure Legends

Figure S1. Schematic representation of the transformation module, the DNA fragments for transforming WT *S. pombe* cells to generate *SPBPJ4664.02*Δ.

Figure S2. The electrophoretic gel image of transformation module (DNA fragments targeting to *SPBPJ4664.02*) used for generating *SPBPJ4664.02*Δ mutant of fission yeast based on homologous recombination. M indicates DL2000 DNA ladder; S1–S6 indicate the products of PCR amplification using 10× diluted pFA6a-KanMX6 DNAs as the respective template.

Figure S3. The molecular characterization via the colony PCR of the 3 transformants grown on the selective YE5S/G418 plates. M indicates DL2000 ladder; S1–S3 indicate the products of PCR amplification using gDNA derived from the transformants 1–3 as the template, respectively.

Figure S4. Schematic representation of recombinant vector pSGP572a-*flo11*, the construct of which involves the insert of *flo11* into the *BglII* and *NotI* sites of pSGP572a, a *E. coli–S. pombe* shuttle vector harboring *Pnmt*, *gfp*, *Ura4*$^{+}$ and pUC119.

Figure S5： Effects of different concentrations of IAA on growth of *C. albicans.* The asterisk * indicated significant differences ($p < 0.05$) using Student's T test.

Figure S6： Effects of different concentrations of dodecanol on growth of *C. albicans*. The asterisk * indicated significant differences ($p < 0.05$) using Student's T test.

Figure S7: Melting curve for *act1, hwp1, ece1, als3* genes during the qPCR analysis.

Supplemental Figures and Tables

Table S1. The PCR primers for generating deletion mutant used in this study.

Application	Name of primers	Sequence (5′-3′)
**PCR-based deletion of gene *SPBPJ4664.02***	F_*SPBPJ4664.02* deletion making	5′ATGAATATATTTTGAAAACAAACTTTCTTGCCTAATCAAAGGATCAACCGATCTGTGCCAACTTTAATGGTGTCTAAGACGGATCCCCGGGTTAATTAA3′
	R_*SPBPJ4664.02* deletion making	5′GTAATGACTTTACGGAACACTCTCGTAATGCATTGGAGAATCAAAAGGTAAACATGCATATGCTTCATAATTCATTGTTGAATTCGAGCTCGTTTAAAC3′
**Colony PCR of *SPBPJ4664.02Δ***	F_upstr._*SPBPJ4664.02*	5′TCTGTCGCTTGGATCATAGT3′
	R_*KanMX6*	5′ATCGCGAGCCCATTTATACC3′

Table S2. qPCR primer sequence.

Gene	Sequence (5′-3′)	PCR product size (bp)	Tm (°C)
hwp1	5′-TGGCTAGTGAAACCTCACC-3′	150	55
	5′-GTTGCATGAGTGGAACTGATTC-3′		56
ece1	5′- CCAGAAATTGTTGCTCGTGTTG -3′	138	56
	5′-CAGGACGCCATCAAAAACG -3′		55
als3	5′-GTGATGCTGGATCTAACGGTATTG-3′	112	57
	5′- GTCTTAGTTTTGTCGCGGTTAGG -3′		57
act1	5′-TTGACCAAACCACTTTCAACTC-3′	152	55
	5′-AGAAGATGGAGCCAAAGCAG-3′		56

Figure S1

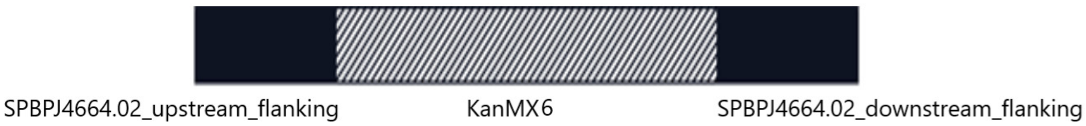

Figure S2

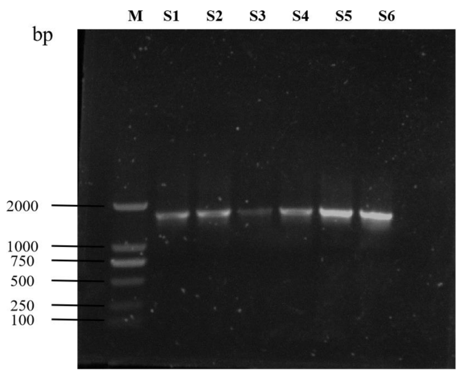

Figure S3

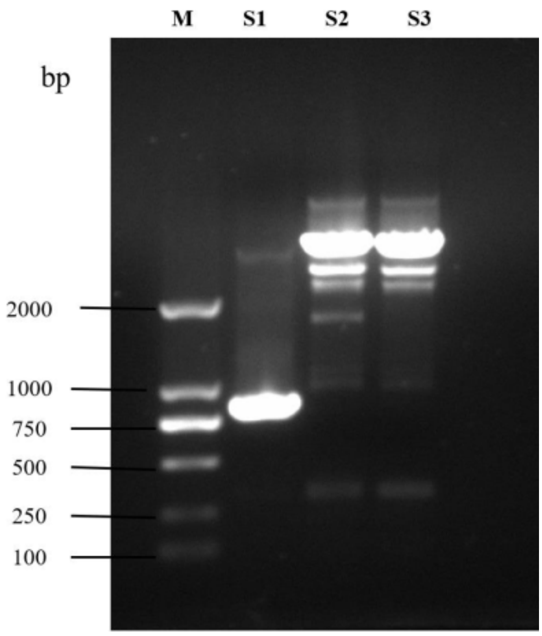

Figure S4

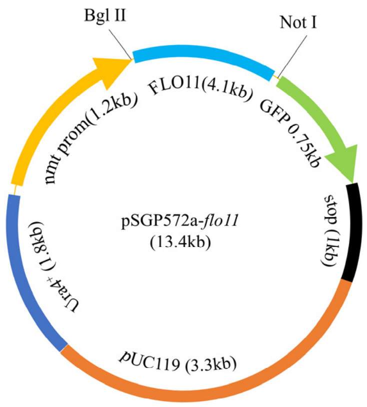

Figure S5

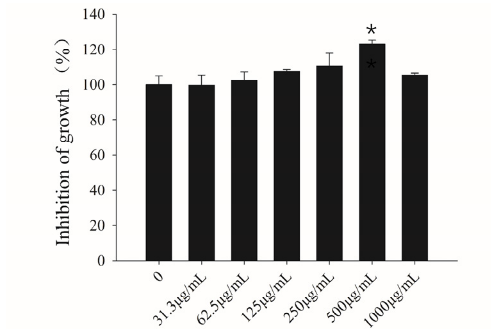

Figure S6

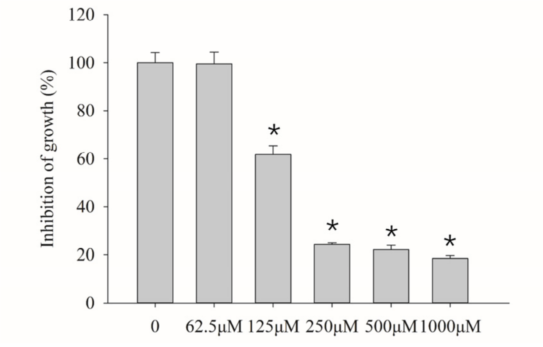

Figure S7

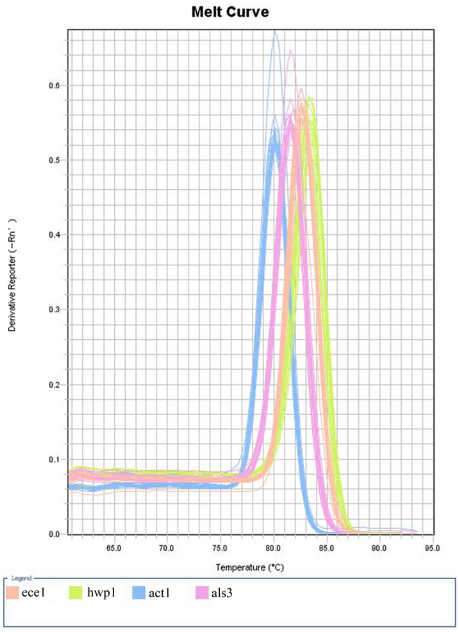

Supplemental Methods

Construction of the *SPBPJ4664.02Δ* deletion mutant

PCR amplification of DNA fragments containing *SPBPJ4664.02* flanking nucleotide sequences for transformation (transformation module)

The homologous recombination technique was employed to generate the *SPBPJ4664.02Δ* knockout mutant, in which the transformation module (Figure S1) was introduced into the WT fission yeast cell targeting the chromosomal locus of *SPBPJ4664.02*, via PCR-based gene targeting (Bähler *et al*., 1998). After homologous recombination of this above-described transformation module and WT genomic DNA, the resulting yeast transformants would display G418 resistance.

As for PCR amplification of this transformation module targeting the gene *SPBPJ4664.02*, plasmid DNA of pFA6a-KanMX6 was used as a template. The primers of 100 nucleotide (nt.) each were designed containing a *SPBPJ4664.02*-specific nucleotide region of homology to the yeast genome (~80 nt.) and a constant priming region corresponding to the plasmid (~20 nt.) based on the principle described by Bähler et al. (1998) and synthesized by Sangon Co. (Shanghai, China). The primers are shown in Table S1. The PCR parameters are as follows: pre-denaturing at 94℃ for 3 min., 30 cycles of denaturing at 94℃ for 30 sec., annealing at 58℃ for 30 sec., extension at 72℃ for 30 sec., followed by a final extension at 72℃ for 5 min. After examination by gel electrophoresis, PCR products from 3~ 4 reactions (tubes) were pooled and the desired DNA fragment was extracted by an equal volume of phenol: chloroform: isoamyl alcohol (25:24:1, v/v), precipitated with ethanol, and dissolved in 10 μL of TE (pH 8.0) for the follow-up transformation.

Transformation of WT *S. pombe* and identification of G418-resistant *SPBPJ4664.02Δ* transformants

The transformation module was introduced into *S. pombe* cells using a protocol by Bähler *et al*. (1998). Briefly, an appropriate volume of WT cells was inoculated from *S. pombe* pre-culture into 30 mL of YE5S liquid and cultivated on a shaker at 200rpm, 30℃ until cell density reached *c.a.* 1 × 10^7cells /mL. The cells were harvested by centrifugation (1,200 ×g, 3 min.), followed by washing in 30 mL of sterile water and in 1 mL of lithium acetate (LiAc)/TE (0.1 M

LiAc, 0.01M Tris–HCl, 1mM EDTA, pH 7.5). The cell pellet was then resuspended in LiAc/TE to give a final concentration of 2×10^9 cells/mL. The concentrated cells of 100 μL were mixed with 2 μL sheared salmon testes DNA (10 mg/mL, Huayueyang Co., Beijing, China) and 10 μL of the transforming DNA which was obtained in the previous step. After 10 min incubation at room temperature, 2.33 volume of 40% PEG 4000 were added. The cell suspension was gently mixed and incubated for 45~ 60 min at 30℃. Following the addition of 43 μL of DMSO, the cells were heat-shocked at 42 ℃ for 5 min. Cells transformed with the transformation module carrying the kanMX6 marker were subsequently washed once with 1 mL of sterile water, resuspended in 0.5 mL of sterile water, and plated onto two YE5S agar plates (250 μL per plate). These plates were incubated for 18 h at 30℃, giving rise to a lawn of cells. The cells were then replica plated onto YE5S plates containing 100 μg/mL G418 (viz. Geneticin, Shanghai Sangon Co., China). The replica plates were incubated for 2–3 d at 30 ℃, and large colonies were restreaked onto fresh YE5S plates containing G418 (YE5S/G418).
The positive colonies (transformants) were chosen on YE5S/G418 plates and transferred to the YE5S/G418 broth for 10~12 h shaking growth at 200rpm, 30℃. The genomic DNAs were extracted from the cells collected from the above culture using TIANamp Yeast DNA Kit (Tiangen Co., Beijing, China) according to the manufacturers' instructions. The colony PCR identification was carried out using the genomic DNA of each colony as the template and the primers F_upstr._SPBPJ4664.02 and R_KanMX6 (Table S1) with PCR thermal parameters as follows: pre-denaturing at 94℃ for 3 min., 30 cycles of 94℃ for 30 sec., annealing at 49℃ for 30 sec., extension at 72℃ for 1 min., followed by the final extension at 72℃ for 5 min. The colony PCR products were subjected to 1% agarose electrophoresis to verify the expected band (~ 900bp).

Effects of IAA and dodecanol on *C. albicans* growth

To detect the effects of IAA and dodecanol on growth of planktonic *C. albicans*, cell suspension of 2mL (10^5CFU/mL) in YPD was added to each 10mL glass tube. Then IAA was added to tubes ranging from 1000 μg/mL and serially diluted to 31.3 μg/mL; Dodecanol was added to tubes ranging from 1000 μM and serially diluted to 62.5 μM (Kimani *et al*., 2021). The cell suspension without compounds was set as the control. The tubes were cultivated in the shaker at 200rpm, 37 ℃ for 48 h. The absorbance of cell suspension was measured at

600nm using colorimetry (PHILES, China). All measurements were performed in triplicates.

Supplemental Results

Construct of fission yeast *SPBPJ4664.02*Δ mutant

The amplification of the transformation module used for generating *SPBPJ4664.02*Δ

The PCR amplification of the transformation module was conducted with the primers as previously described in Table S1 of the SI, using 1× or 10× diluted pFA6a-KanMX6 DNAs as the respective template to ensure the optimized amplification results. The PCR products were subjected to agarose electrophoresis examination, where the desired DNA fragments should be of 1515bp length. Figure S2 illustrated that all 10× diluted pFA6a-KanMX6 DNA as the template might generate satisfactory results, evidenced by the bands as expected.

Construction of fission yeast *SPBPJ4664.02*Δ mutant and molecular characterization

The WT fission yeast cells were transformed with the above-mentioned DNA fragments targeting to *SPBPJ4664.02* (the so-called transformation module), based on the protocol by Bähler et al. (1998). Eventually, three transformants were grown on the YE5S/G418 selective plates, which were subjected to colony PCR identification using the forward primer namely F_upstr._SPBPJ4664.02 corresponding to ~500bp upstream sequences flanking the gene of interest (*SPBPJ4664.02* herein), and reverse primer namely R_KanMX6 corresponding to the sequence within the KanM6 marker of pFA6a-KanM6. The colony PCR products were examined by agarose (0.85%) electrophoresis and photographed (Figure S3). According to the afore-described primers for colony PCR, the expected band would be of 875 bp length (Figure S3). As shown in Figure S3, the colony #1 was verified to be *SPBPJ4664.02*Δ, evidenced by the expected band on the gel, indicating that the *SPBPJ4664.02* gene on the genomic DNA had been swapped with the KanMX6 via homologous recombination.

Impacts of IAA and dodecanol on *C. albicans* growth

IAA and dodecanol were assayed for their ability to inhibit the planktonic growth of *C. albicans* at 37℃. In these tests, growths of *C. albicans* were studied in the presence of IAA ranging from 31.3 to 1000 μg/mL (Figure S5), and dodecanol of 62.5 to 1000μM (Figure S6), respectively. As shown in Figure S5, IAA seemed not to inhibit cell growth, but it showed a slightly promotive effects at the concentrations of 500 μg/mL. In contrast, dodecanol displayed a potent inhibitory effect on growth at the concentrations ranging from 125 μM to 1000 μM, without affecting cell

viability (Figure S6).

Supplemental References

Bähler, J.; Wu, J.Q.; Longtine, M.S.; Shah, N. G.; McKenzie, A.; Steever, A. B.; Wach, A.; Philippsen, P.; Pringle, J. R. Heterologous modules for efficient and versatile PCR-based gene targeting in *Schizosaccharomyces pombe. Yeast* **1998**, *14*, 943–951.

Kimani, B.J.; Kerekes, E.B.; Szebenyi, C.; Krisch, J.; Vágvölgyi, C.; Papp, T.; Takó, M. In vitro activity of selected phenolic compounds against planktonic and biofilm cells of food-contaminating yeasts. *Foods* **2021**, *10*, 1652.
